# 4-(2-Carboxy­benzo­yl)benzoic acid–4,4′-bipyridine (1/1)

**DOI:** 10.1107/S1600536808037823

**Published:** 2008-11-20

**Authors:** Jian-Ge Wang, Jian-Hua Qin

**Affiliations:** a College of Chemistry and Chemical Engineering, Luoyang Normal University, Luoyang 471022, People’s Republic of China

## Abstract

In the heteromolecular title compound, C_15_H_10_O_5_·C_10_H_8_N_2_, the two components are linked by O—H⋯N hydrogen bonds to form four-component ring supra­molecular assemblies. These are further inter­connected with neighbouring mol­ecules by weak inter­molecular C—H⋯π inter­actions and C—H⋯O hydrogen bonds to generate a three-dimensional network.

## Related literature

For details of the C—H⋯O hydrogen bond, see: Bhogala *et al.* (2005 ▶); Wang *et al.* (2008 ▶). For details of the C—H⋯π inter­action, see: Fun & Kia (2008 ▶).
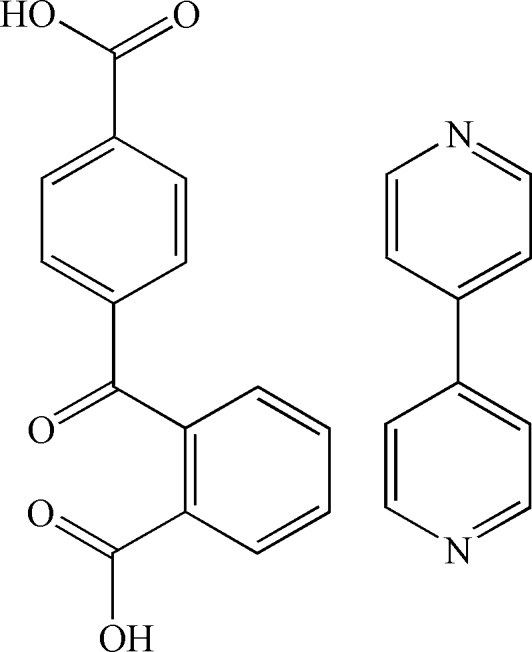


## Experimental

### 

#### Crystal data

C_15_H_10_O_5_·C_10_H_8_N_2_
*M* *_r_* = 426.41
Monoclinic, 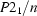

*a* = 7.6883 (6) Å
*b* = 24.1886 (18) Å
*c* = 10.9560 (8) Å
β = 95.873 (1)°
*V* = 2026.8 (3) Å^3^
*Z* = 4
Mo *K*α radiation
μ = 0.10 mm^−1^
*T* = 296 (2) K
0.35 × 0.28 × 0.16 mm

#### Data collection

Bruker CCD area detector diffractometer
Absorption correction: multi-scan (*SADABS*; Bruker, 1997 ▶) *T*_min_ = 0.957, *T*_max_ = 0.984
10190 measured reflections
3607 independent reflections
2177 reflections with *I* > 2σ(*I*)
*R*_int_ = 0.041

#### Refinement

*R*[*F* ^2^ > 2σ(*F* ^2^)] = 0.045
*wR*(*F* ^2^) = 0.128
*S* = 1.02
3607 reflections
292 parameters
H-atom parameters constrained
Δρ_max_ = 0.21 e Å^−3^
Δρ_min_ = −0.17 e Å^−3^

### 

Data collection: *SMART* (Bruker, 1997 ▶); cell refinement: *SAINT* (Bruker, 1997 ▶); data reduction: *SAINT*; program(s) used to solve structure: *SHELXS97* (Sheldrick, 2008 ▶); program(s) used to refine structure: *SHELXL97* (Sheldrick, 2008 ▶); molecular graphics: *SHELXTL* (Sheldrick, 2008 ▶); software used to prepare material for publication: *SHELXTL*.

## Supplementary Material

Crystal structure: contains datablocks I, global. DOI: 10.1107/S1600536808037823/si2130sup1.cif

Structure factors: contains datablocks I. DOI: 10.1107/S1600536808037823/si2130Isup2.hkl

Additional supplementary materials:  crystallographic information; 3D view; checkCIF report

## Figures and Tables

**Table 1:** Hydrogen-bond geometry (Å, °) *Cg*1 is the centroid of the C19–C24 ring.

*D*—H⋯*A*	*D*—H	H⋯*A*	*D*⋯*A*	*D*—H⋯*A*
C8—H8⋯O4^i^	0.93	2.51	3.282 (3)	141
O5—H5⋯N2^ii^	0.82	1.84	2.641 (2)	166
O2—H2⋯N1^iii^	0.82	1.79	2.595 (2)	168
C1—H1⋯*Cg*1^iv^	0.93	2.54	3.449 (3)	165

Symmetry codes: (i) 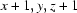; (ii) 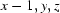; (iii) 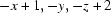; (iv) 
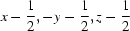
.

**Fig. 1.**
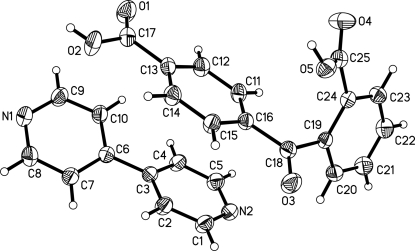
A view of the heteromolecular components of the title compound with the atom labelling scheme. Displacement ellipsoids are drawn at the 30% probability level.

**Fig. 2.**
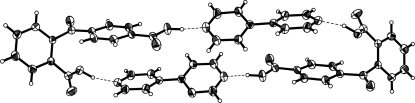
A view of the four-component ring supramolecular adducts, formed by O—H···N hydrogen bonds.

**Fig. 3.**
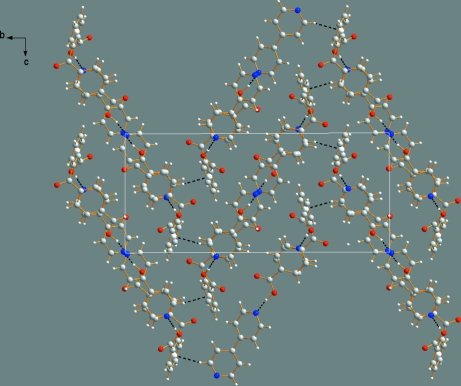
A packing section of the title compound viewed down the a axis, indicating the C—H···π contacts by dashed lines.

**Fig. 4.**
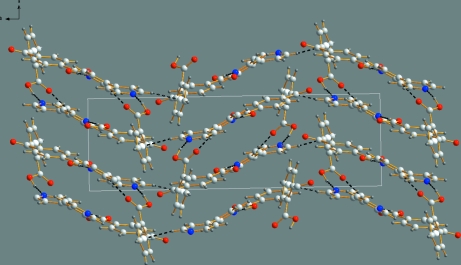
A view of the molecular packing down the c axis.

## supplementary crystallographic information

### Comment

The asymmetric unit consists of one 4,4'-bipyridine molecule and one 4-(2-carboxylbenzoyl)benzoic acid molecule (Fig. 1). The two components are linked by O—H···N hydrogen bonds to form four-component ring supramolecular adducts (Tab. 1 & Fig. 2). The four-component ring supramolecular adducts interact with neigboring molecules *via* weak intermolecular C—H···π interactions and C—H···O hydrogen bonds (Tab. 1). The C—H···π interactions link the four-component ring supramolecular adducts into two-dimensional layers (Fig. 3), which are further connected by weak intermolecular C—H···O hydrogen bonds to generate a three-dimensional supramolecular structure (Fig. 4).

### Experimental

4,4'-bipyridine (0.1 mmol), 4-(2-carboxylbenzoyl)benzoic acid (0.1 mmol), and NaOH (0.2 mmol) were added to a H_2_O solution (15 ml) in a Teflon-lined stainless steel reactor. The mixture was heated at 473 K for 3 d, and then slowly cooled down to room temperature. Colorless crystals of the title compound were obtained.

### Refinement

All hydrogen atoms were positioned geometrically and treated as riding, with C—H bond lengths constrained to 0.93 Å (aromatic CH) and O—H bond lengths constrained to 0.82 Å (OH), and with *U*_iso_(H) = 1.2*U*_eq_(C) and *U*_iso_(H) = 1.5*U*_eq_(O).

### Crystal data

**Table:**

C_15_H_10_O_5_·C_10_H_8_N_2_	*F*_000_ = 888
*M**_r_* = 426.41	*D*_x_ = 1.397 Mg m^−^^3^
Monoclinic, *P*2_1_/*n*	Mo *K*α radiation λ = 0.71073 Å
Hall symbol: -P2yn	Cell parameters from 1385 reflections
*a* = 7.6883 (6) Å	θ = 2.5–21.2º
*b* = 24.1886 (18) Å	µ = 0.10 mm^−^^1^
*c* = 10.9560 (8) Å	*T* = 296 (2) K
β = 95.8730 (10)º	Block, colorless
*V* = 2026.8 (3) Å^3^	0.35 × 0.28 × 0.16 mm
*Z* = 4	

### Data collection

**Table:**

Bruker CCD area detector diffractometer	3607 independent reflections
Radiation source: fine-focus sealed tube	2177 reflections with *I* > 2σ(*I*)
Monochromator: graphite	*R*_int_ = 0.041
*T* = 296(2) K	θ_max_ = 25.1º
φ and ω scans	θ_min_ = 1.7º
Absorption correction: multi-scan(SADABS; Bruker, 1997)	*h* = −9→9
*T*_min_ = 0.957, *T*_max_ = 0.984	*k* = −28→28
10190 measured reflections	*l* = −13→7

### Refinement

**Table:**

Refinement on *F*^2^	Hydrogen site location: inferred from neighbouring sites
Least-squares matrix: full	H-atom parameters constrained
*R*[*F*^2^ > 2σ(*F*^2^)] = 0.045	*w* = 1/[σ^2^(*F*_o_^2^) + (0.060*P*)^2^] where *P* = (*F*_o_^2^ + 2*F*_c_^2^)/3
*wR*(*F*^2^) = 0.128	(Δ/σ)_max_ < 0.001
*S* = 1.02	Δρ_max_ = 0.21 e Å^−^^3^
3607 reflections	Δρ_min_ = −0.17 e Å^−^^3^
292 parameters	Extinction correction: SHELXL, Fc^*^=kFc[1+0.001xFc^2^λ^3^/sin(2θ)]^-1/4^
Primary atom site location: structure-invariant direct methods	Extinction coefficient: 0.0131 (14)
Secondary atom site location: difference Fourier map	

### Fractional atomic coordinates and isotropic or equivalent isotropic displacement parameters (Å^2^)

**Table:**

	*x*	*y*	*z*	*U*_iso_*/*U*_eq_	
N1	0.7560 (3)	0.00682 (8)	0.99002 (17)	0.0561 (5)	
N2	0.9586 (2)	0.15564 (8)	0.46655 (17)	0.0512 (5)	
O1	0.2912 (3)	−0.00260 (8)	0.69911 (15)	0.0779 (6)	
O2	0.3035 (3)	0.06365 (7)	0.84053 (15)	0.0671 (5)	
H2	0.2837	0.0379	0.8859	0.101*	
O3	0.5482 (2)	0.25808 (7)	0.42728 (14)	0.0659 (5)	
O4	0.0942 (2)	0.13238 (8)	0.19022 (17)	0.0789 (6)	
O5	0.1584 (2)	0.20365 (7)	0.31530 (14)	0.0532 (4)	
H5	0.0822	0.1908	0.3541	0.080*	
C1	0.9364 (3)	0.18081 (10)	0.5716 (2)	0.0557 (7)	
H1	0.9493	0.2190	0.5754	0.067*	
C2	0.8955 (3)	0.15357 (10)	0.6751 (2)	0.0530 (6)	
H2A	0.8810	0.1733	0.7463	0.064*	
C3	0.8761 (3)	0.09687 (9)	0.67290 (19)	0.0426 (5)	
C4	0.8951 (3)	0.07078 (10)	0.5629 (2)	0.0521 (6)	
H4	0.8804	0.0327	0.5560	0.062*	
C5	0.9359 (3)	0.10128 (11)	0.4632 (2)	0.0556 (6)	
H5A	0.9480	0.0828	0.3901	0.067*	
C6	0.8351 (3)	0.06565 (9)	0.7835 (2)	0.0436 (6)	
C7	0.8857 (3)	0.08447 (10)	0.9015 (2)	0.0515 (6)	
H7	0.9477	0.1174	0.9134	0.062*	
C8	0.8436 (3)	0.05426 (11)	1.0008 (2)	0.0571 (7)	
H8	0.8781	0.0677	1.0791	0.069*	
C9	0.7071 (3)	−0.01128 (10)	0.8773 (2)	0.0618 (7)	
H9	0.6445	−0.0442	0.8682	0.074*	
C10	0.7445 (3)	0.01621 (10)	0.7733 (2)	0.0561 (7)	
H10	0.7089	0.0016	0.6962	0.067*	
C11	0.4552 (3)	0.11251 (9)	0.44658 (19)	0.0472 (6)	
H11	0.4816	0.1016	0.3692	0.057*	
C12	0.4068 (3)	0.07337 (9)	0.5287 (2)	0.0503 (6)	
H12	0.4006	0.0363	0.5060	0.060*	
C13	0.3673 (3)	0.08886 (9)	0.64455 (19)	0.0441 (6)	
C14	0.3797 (3)	0.14392 (10)	0.6772 (2)	0.0497 (6)	
H14	0.3544	0.1548	0.7548	0.060*	
C15	0.4293 (3)	0.18291 (9)	0.59573 (19)	0.0481 (6)	
H15	0.4392	0.2198	0.6194	0.058*	
C16	0.4644 (3)	0.16755 (9)	0.47894 (19)	0.0416 (5)	
C17	0.3161 (3)	0.04481 (11)	0.7299 (2)	0.0518 (6)	
C18	0.5073 (3)	0.21165 (10)	0.3914 (2)	0.0455 (6)	
C19	0.5114 (3)	0.19699 (9)	0.25883 (18)	0.0408 (5)	
C20	0.6710 (3)	0.20203 (9)	0.2104 (2)	0.0499 (6)	
H20	0.7674	0.2158	0.2592	0.060*	
C21	0.6878 (3)	0.18689 (10)	0.0910 (2)	0.0561 (6)	
H21	0.7957	0.1898	0.0602	0.067*	
C22	0.5460 (3)	0.16750 (10)	0.0174 (2)	0.0565 (7)	
H22	0.5575	0.1576	−0.0634	0.068*	
C23	0.3861 (3)	0.16268 (9)	0.0633 (2)	0.0499 (6)	
H23	0.2902	0.1496	0.0129	0.060*	
C24	0.3669 (3)	0.17700 (9)	0.18334 (19)	0.0419 (5)	
C25	0.1923 (3)	0.16868 (10)	0.2289 (2)	0.0491 (6)	

### Atomic displacement parameters (Å^2^)

**Table:**

	*U*^11^	*U*^22^	*U*^33^	*U*^12^	*U*^13^	*U*^23^
N1	0.0681 (14)	0.0556 (14)	0.0461 (12)	0.0026 (11)	0.0131 (10)	0.0071 (10)
N2	0.0492 (12)	0.0565 (14)	0.0486 (12)	−0.0050 (10)	0.0086 (9)	0.0031 (10)
O1	0.1202 (17)	0.0535 (12)	0.0602 (12)	−0.0246 (11)	0.0108 (10)	0.0006 (10)
O2	0.1031 (15)	0.0560 (11)	0.0451 (10)	−0.0019 (10)	0.0217 (10)	0.0098 (8)
O3	0.0921 (14)	0.0547 (12)	0.0515 (11)	−0.0205 (10)	0.0106 (9)	−0.0029 (9)
O4	0.0604 (12)	0.0931 (15)	0.0854 (14)	−0.0297 (11)	0.0174 (10)	−0.0236 (11)
O5	0.0504 (10)	0.0576 (11)	0.0538 (11)	−0.0020 (8)	0.0153 (8)	0.0034 (8)
C1	0.0671 (17)	0.0449 (15)	0.0563 (17)	−0.0067 (12)	0.0129 (13)	0.0014 (12)
C2	0.0642 (16)	0.0476 (15)	0.0489 (15)	−0.0051 (12)	0.0143 (12)	−0.0005 (11)
C3	0.0406 (13)	0.0469 (14)	0.0412 (13)	−0.0016 (10)	0.0077 (10)	0.0003 (11)
C4	0.0633 (16)	0.0447 (14)	0.0505 (15)	−0.0066 (12)	0.0167 (12)	−0.0025 (11)
C5	0.0612 (16)	0.0587 (17)	0.0487 (15)	−0.0053 (13)	0.0149 (12)	−0.0048 (12)
C6	0.0427 (13)	0.0447 (14)	0.0439 (13)	0.0025 (10)	0.0063 (10)	0.0029 (11)
C7	0.0580 (15)	0.0504 (15)	0.0466 (15)	−0.0050 (12)	0.0083 (12)	0.0004 (12)
C8	0.0671 (17)	0.0623 (18)	0.0425 (14)	0.0015 (14)	0.0075 (12)	0.0006 (13)
C9	0.0805 (19)	0.0502 (16)	0.0550 (16)	−0.0103 (13)	0.0090 (14)	0.0087 (13)
C10	0.0767 (18)	0.0499 (16)	0.0413 (14)	−0.0090 (13)	0.0050 (12)	0.0010 (11)
C11	0.0587 (15)	0.0484 (15)	0.0351 (12)	0.0046 (12)	0.0070 (11)	−0.0012 (11)
C12	0.0643 (16)	0.0406 (14)	0.0457 (14)	0.0020 (11)	0.0039 (12)	0.0001 (11)
C13	0.0472 (14)	0.0500 (15)	0.0347 (13)	0.0023 (11)	0.0018 (10)	0.0037 (11)
C14	0.0601 (15)	0.0511 (15)	0.0387 (13)	0.0014 (12)	0.0092 (11)	−0.0031 (11)
C15	0.0606 (15)	0.0421 (14)	0.0421 (14)	−0.0018 (11)	0.0068 (11)	0.0010 (11)
C16	0.0427 (13)	0.0462 (14)	0.0360 (12)	0.0002 (10)	0.0044 (10)	0.0022 (10)
C17	0.0573 (16)	0.0556 (17)	0.0418 (15)	−0.0004 (13)	0.0019 (12)	0.0065 (12)
C18	0.0449 (14)	0.0508 (16)	0.0409 (13)	−0.0018 (11)	0.0054 (10)	0.0017 (11)
C19	0.0438 (13)	0.0427 (13)	0.0365 (12)	−0.0013 (10)	0.0067 (10)	0.0052 (10)
C20	0.0435 (14)	0.0578 (16)	0.0485 (14)	−0.0035 (11)	0.0052 (11)	0.0032 (12)
C21	0.0468 (15)	0.0667 (17)	0.0566 (16)	0.0037 (12)	0.0144 (12)	0.0023 (13)
C22	0.0624 (17)	0.0642 (17)	0.0448 (14)	0.0070 (13)	0.0150 (13)	−0.0036 (12)
C23	0.0516 (15)	0.0510 (15)	0.0461 (14)	−0.0025 (11)	0.0012 (11)	−0.0041 (11)
C24	0.0458 (14)	0.0396 (13)	0.0408 (13)	0.0003 (10)	0.0064 (11)	0.0046 (10)
C25	0.0494 (15)	0.0539 (16)	0.0439 (14)	−0.0026 (12)	0.0041 (11)	0.0024 (12)

### Geometric parameters (Å, °)

**Table:**

N1—C9	1.328 (3)	C9—H9	0.9300
N1—C8	1.330 (3)	C10—H10	0.9300
N2—C5	1.327 (3)	C11—C16	1.378 (3)
N2—C1	1.328 (3)	C11—C12	1.383 (3)
O1—C17	1.205 (3)	C11—H11	0.9300
O2—C17	1.308 (3)	C12—C13	1.387 (3)
O2—H2	0.8200	C12—H12	0.9300
O3—C18	1.220 (3)	C13—C14	1.380 (3)
O4—C25	1.205 (3)	C13—C17	1.497 (3)
O5—C25	1.315 (3)	C14—C15	1.379 (3)
O5—H5	0.8200	C14—H14	0.9300
C1—C2	1.376 (3)	C15—C16	1.385 (3)
C1—H1	0.9300	C15—H15	0.9300
C2—C3	1.379 (3)	C16—C18	1.493 (3)
C2—H2A	0.9300	C18—C19	1.499 (3)
C3—C4	1.382 (3)	C19—C20	1.391 (3)
C3—C6	1.489 (3)	C19—C24	1.402 (3)
C4—C5	1.380 (3)	C20—C21	1.377 (3)
C4—H4	0.9300	C20—H20	0.9300
C5—H5A	0.9300	C21—C22	1.371 (3)
C6—C10	1.383 (3)	C21—H21	0.9300
C6—C7	1.388 (3)	C22—C23	1.380 (3)
C7—C8	1.376 (3)	C22—H22	0.9300
C7—H7	0.9300	C23—C24	1.383 (3)
C8—H8	0.9300	C23—H23	0.9300
C9—C10	1.375 (3)	C24—C25	1.493 (3)
			
C9—N1—C8	117.3 (2)	C14—C13—C12	118.8 (2)
C5—N2—C1	116.7 (2)	C14—C13—C17	122.7 (2)
C17—O2—H2	109.5	C12—C13—C17	118.4 (2)
C25—O5—H5	109.5	C15—C14—C13	120.6 (2)
N2—C1—C2	123.7 (2)	C15—C14—H14	119.7
N2—C1—H1	118.1	C13—C14—H14	119.7
C2—C1—H1	118.1	C14—C15—C16	120.5 (2)
C1—C2—C3	119.7 (2)	C14—C15—H15	119.8
C1—C2—H2A	120.2	C16—C15—H15	119.8
C3—C2—H2A	120.2	C11—C16—C15	119.2 (2)
C2—C3—C4	116.7 (2)	C11—C16—C18	122.3 (2)
C2—C3—C6	121.4 (2)	C15—C16—C18	118.6 (2)
C4—C3—C6	121.9 (2)	O1—C17—O2	124.5 (2)
C5—C4—C3	119.9 (2)	O1—C17—C13	123.2 (2)
C5—C4—H4	120.0	O2—C17—C13	112.3 (2)
C3—C4—H4	120.0	O3—C18—C16	121.2 (2)
N2—C5—C4	123.2 (2)	O3—C18—C19	119.9 (2)
N2—C5—H5A	118.4	C16—C18—C19	118.8 (2)
C4—C5—H5A	118.4	C20—C19—C24	118.8 (2)
C10—C6—C7	116.7 (2)	C20—C19—C18	117.1 (2)
C10—C6—C3	121.3 (2)	C24—C19—C18	124.02 (18)
C7—C6—C3	122.0 (2)	C21—C20—C19	120.7 (2)
C8—C7—C6	119.7 (2)	C21—C20—H20	119.6
C8—C7—H7	120.1	C19—C20—H20	119.6
C6—C7—H7	120.1	C22—C21—C20	120.2 (2)
N1—C8—C7	123.1 (2)	C22—C21—H21	119.9
N1—C8—H8	118.4	C20—C21—H21	119.9
C7—C8—H8	118.4	C21—C22—C23	120.0 (2)
N1—C9—C10	123.3 (2)	C21—C22—H22	120.0
N1—C9—H9	118.4	C23—C22—H22	120.0
C10—C9—H9	118.4	C22—C23—C24	120.7 (2)
C9—C10—C6	119.8 (2)	C22—C23—H23	119.6
C9—C10—H10	120.1	C24—C23—H23	119.6
C6—C10—H10	120.1	C23—C24—C19	119.49 (19)
C16—C11—C12	120.3 (2)	C23—C24—C25	118.1 (2)
C16—C11—H11	119.9	C19—C24—C25	122.33 (19)
C12—C11—H11	119.9	O4—C25—O5	124.0 (2)
C11—C12—C13	120.6 (2)	O4—C25—C24	122.1 (2)
C11—C12—H12	119.7	O5—C25—C24	113.9 (2)
C13—C12—H12	119.7		
			
C5—N2—C1—C2	1.3 (3)	C14—C15—C16—C18	176.4 (2)
N2—C1—C2—C3	0.2 (4)	C14—C13—C17—O1	173.8 (2)
C1—C2—C3—C4	−1.7 (3)	C12—C13—C17—O1	−7.4 (3)
C1—C2—C3—C6	178.9 (2)	C14—C13—C17—O2	−6.8 (3)
C2—C3—C4—C5	1.6 (3)	C12—C13—C17—O2	172.1 (2)
C6—C3—C4—C5	−179.0 (2)	C11—C16—C18—O3	−165.6 (2)
C1—N2—C5—C4	−1.5 (3)	C15—C16—C18—O3	15.8 (3)
C3—C4—C5—N2	0.0 (4)	C11—C16—C18—C19	10.3 (3)
C2—C3—C6—C10	151.9 (2)	C15—C16—C18—C19	−168.35 (19)
C4—C3—C6—C10	−27.6 (3)	O3—C18—C19—C20	59.3 (3)
C2—C3—C6—C7	−28.2 (3)	C16—C18—C19—C20	−116.6 (2)
C4—C3—C6—C7	152.4 (2)	O3—C18—C19—C24	−123.0 (2)
C10—C6—C7—C8	−0.5 (3)	C16—C18—C19—C24	61.1 (3)
C3—C6—C7—C8	179.6 (2)	C24—C19—C20—C21	−0.9 (3)
C9—N1—C8—C7	−0.4 (4)	C18—C19—C20—C21	176.9 (2)
C6—C7—C8—N1	0.3 (4)	C19—C20—C21—C22	1.1 (4)
C8—N1—C9—C10	0.7 (4)	C20—C21—C22—C23	−0.5 (4)
N1—C9—C10—C6	−0.9 (4)	C21—C22—C23—C24	−0.3 (4)
C7—C6—C10—C9	0.7 (3)	C22—C23—C24—C19	0.4 (3)
C3—C6—C10—C9	−179.3 (2)	C22—C23—C24—C25	−177.6 (2)
C16—C11—C12—C13	−0.2 (3)	C20—C19—C24—C23	0.2 (3)
C11—C12—C13—C14	−0.9 (3)	C18—C19—C24—C23	−177.5 (2)
C11—C12—C13—C17	−179.8 (2)	C20—C19—C24—C25	178.1 (2)
C12—C13—C14—C15	0.4 (3)	C18—C19—C24—C25	0.4 (3)
C17—C13—C14—C15	179.3 (2)	C23—C24—C25—O4	30.4 (3)
C13—C14—C15—C16	1.2 (3)	C19—C24—C25—O4	−147.5 (2)
C12—C11—C16—C15	1.7 (3)	C23—C24—C25—O5	−150.7 (2)
C12—C11—C16—C18	−176.9 (2)	C19—C24—C25—O5	31.3 (3)
C14—C15—C16—C11	−2.2 (3)		

### Hydrogen-bond geometry (Å, °)

**Table:**

*D*—H···*A*	*D*—H	H···*A*	*D*···*A*	*D*—H···*A*
C8—H8···O4^i^	0.93	2.51	3.282 (3)	141
O5—H5···N2^ii^	0.82	1.84	2.641 (2)	166
O2—H2···N1^iii^	0.82	1.79	2.595 (2)	168
C1—H1···Cg1^iv^	0.93	2.54	3.449 (3)	165

Symmetry codes: (i) *x*+1, *y*, *z*+1; (ii) *x*−1, *y*, *z*; (iii) −*x*+1, −*y*, −*z*+2; (iv) *x*−1/2, −*y*−1/2, *z*−1/2.

## References

[bb1] Bhogala, B. R., Basavoju, S. & Nangia, A. (2005). *Cryst. Growth Des.* **5**, 1683–1686.

[bb2] Bruker (1997). *SMART*, *SAINT* and *SADABS* Bruker AXS Inc., Madison, Wisconsin, USA.

[bb3] Fun, H.-K. & Kia, R. (2008). *Acta Cryst.* E**64**, m1116–m1117. 10.1107/S1600536808024306 PMC2960584 21201579

[bb4] Sheldrick, G. M. (2008). *Acta Cryst.* A**64**, 112–122. 10.1107/S0108767307043930 18156677

[bb5] Wang, Y.-T., Tang, G.-M., Zhang, Y.-C. & Wan, W.-Z. (2008). *Acta Cryst.* E**64**, o1753. 10.1107/S1600536808025567 PMC2960605 21201735

